# O050. Chronic daily headache and body mass index: a meta-analysis of observational studies

**DOI:** 10.1186/1129-2377-16-S1-A64

**Published:** 2015-09-28

**Authors:** Cindy Tiseo, Diana Degan, Raffaele Ornello, Amleto Gabriele, Francesca Pistoia, Antonio Carolei, Simona Sacco

**Affiliations:** Department of Neurology, University of L'Aquila, L'Aquila, Italy

## Background

Many studies have investigated the association between chronic daily headache (CDH) and normal weight, pre-obesity, and obesity, with controversial results. A meta-analysis of observational studies was conducted in order to clarify the association between CDH and body mass index (BMI) categories.

## Methods

Studies published up to April 2015 about the association between CDH and BMI were systematically searched from multiple electronic databases. We included in the analysis observational studies in the English language with CDH as outcome variables, and pre-obesity or obesity as compared with normal weight as exposure variables. Only the studies which defined BMI categories according to the World Health Organization criteria for the Western population were included (underweight, <18.5 Kg/m^2^; normal range, 18.5-24.9 Kg/m^2^; overweight, ≥25.0 Kg/m^2^; pre-obesity, 25.0-29.9 Kg/m^2^; class I obesity 30.0-34.9 Kg/m^2^; class II obesity 35.0-39.9 Kg/m^2^; class III obesity ≥40.0 Kg/m^2^). Pooled adjusted effect estimate (PAEE) with 95% confidence interval (CI) was calculated to examine the strength of the association using random-effects models.

## Results

**Figure 1:**
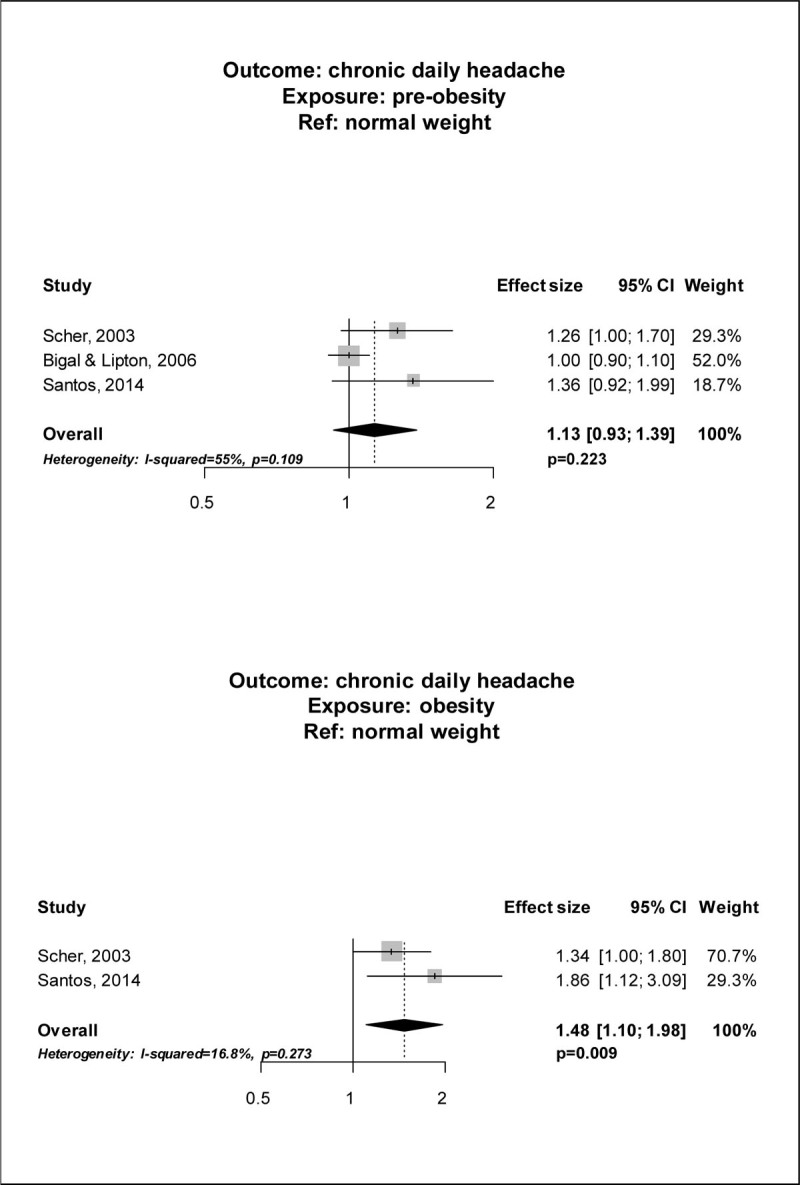

**Figure 2:**
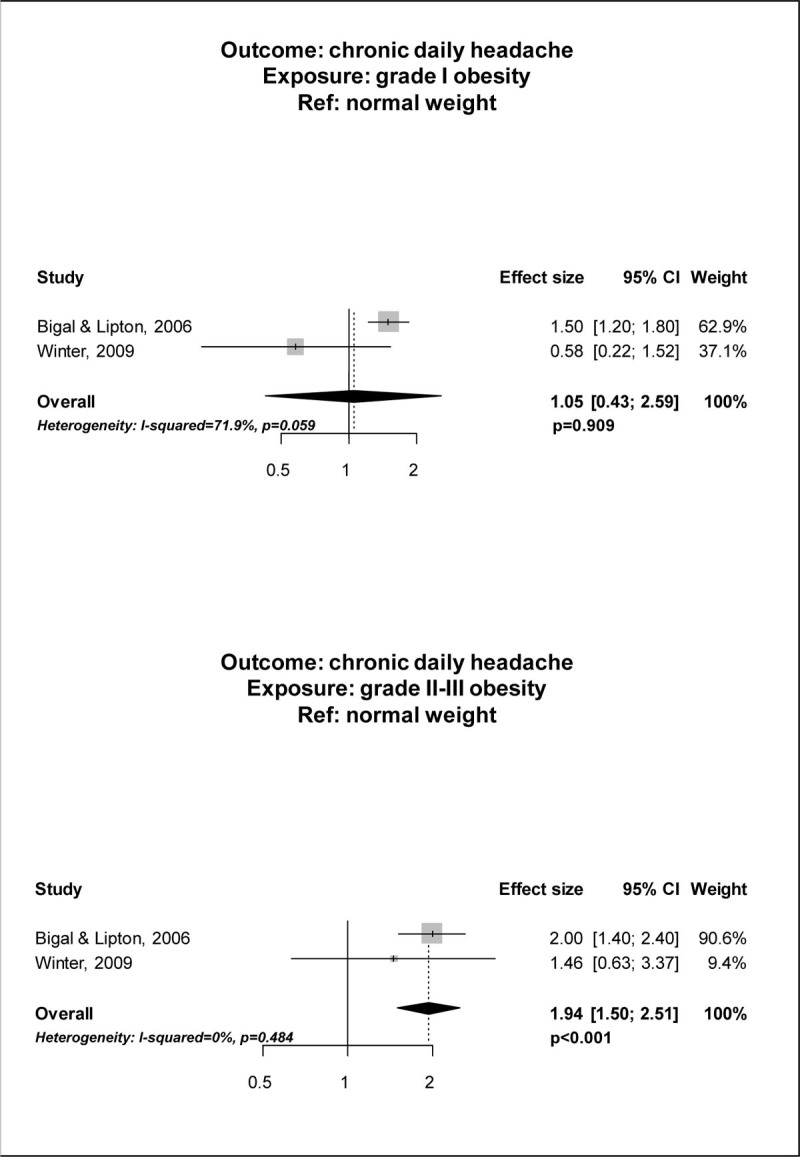

Out of 2,022 records, 4 studies[1–4] met the selection criteria and were included in the meta-analysis. The pooled analysis suggested an increased risk of having CDH in obese subjects (PAEE 1.48; 95% CI, 1.10; 1.98; p = 0.009) as compared to normal weight subjects, while the risk in pre-obese subjects was not different when compared to that of normal weight subjects (PAEE 1.13; 95% CI 0.93-1.39; p = 0.223) (Figure 1). Data analysis according to BMI categories found that subjects with grade II-III obesity had a higher risk of CDH (PAEE 1.94; 95% CI, 1.50-2.51; p < 0.001) than normal weight subjects, while grade I obesity was not associated with a higher risk of CDH (PAEE 1.05; 95% CI 0.43-2.59; p = 0.909) (Figure 2).

## Conclusions

According to this meta-analysis of observational studies there is an association between CDH and moderate and severe obesity. This association suggests that body weight management may be a viable strategy for the prevention of chronification in patients suffering from both migraine and tension-type headache.
